# Post‐kidney transplant cancers: Racial and ethnic differences in sun‐exposed skin versus non‐sun‐exposed anogenital skin

**DOI:** 10.1002/cam4.5431

**Published:** 2022-11-14

**Authors:** Kotaro Takeda, Carolann Risley, Aisha Kousar, Kimberly P. Briley, Karyn Prenshaw, Rajesh Talluri, Kim R. Geisinger, Lorita M. Rebellato

**Affiliations:** ^1^ Department of Pathology and Laboratory Medicine, Brody School of Medicine East Carolina University and Vidant Medical Center Greenville North Carolina USA; ^2^ Department of Cell and Molecular Biology, and Cancer Center and Research Institute University of Mississippi Medical Center, School of Nursing, School of Medicine Jackson Mississippi USA; ^3^ Department of Data Science, School of Population Health University of Mississippi Medical Center Jackson Mississippi USA; ^4^ Walter Reed Military Medical Center The Joint Pathology Center Silver Springs Maryland USA

**Keywords:** cancer, human papillomavirus, kidney transplantation, p16, race, skin

## Abstract

**Background:**

Transplant recipients have a 2‐ to 4‐fold increased risk of developing malignancies over the general population. Cancer is the second most common cause of death for recipients. The magnitude of the risk depends on the cancer type and increases in viral‐related malignancies. Skin cancer is the most common. However, data in most cancer registries is limited to cutaneous melanomas, thereby limiting the epidemiologic examination of cancer risk in non‐melanoma skin cancer. Our goal was to evaluate post‐kidney transplant cancer cases and sites in our population to guide screening recommendations.

**Methods:**

Between 2009 and 2015, a retrospective study of adult kidney recipients transplanted at East Carolina University was conducted. The first cancer diagnosis after transplant through February 18, 2020, was captured and analyzed. Patient demographics, cancer sites, and histological diagnoses were analyzed and compared. p16 immunohistochemistry was used as a surrogate marker for high‐risk human papillomavirus (HPV) infection.

**Results:**

Retrospectively, kidney transplant recipients were analyzed (*N* = 439), the majority were non‐Hispanic Black (NHB) individuals, 312 (71.1%), and 127 (28.9%) were non‐Hispanic White (NHW) individuals. Of these, 59 (13.4%) developed a posttransplant malignancy, with the majority on sun‐exposed skin found in NHW. NHB had all anogenital/mucosa skin cancers on non‐sun‐exposed skin. Of these detected in NHB, all were squamous cell carcinomas, with five out of six (83.3%) being positive for p16.

**Conclusions:**

Posttransplant malignancy differed significantly by race, site, and potential source of etiology. The majority of malignancies are likely explained by acceleration of precursor lesions from prior exposure to ultraviolet rays or HPV.

## Graphical abstract

This study demonstrated that posttransplant skin malignancies differed significantly between non‐Hispanic White and non‐Hispanic Black kidney recipients by cancer site and patient skin tone. Comprehensive full body dermatologic inspection following kidney transplantation is critical and should include close inspection of all skin, including the anal and genital regions.

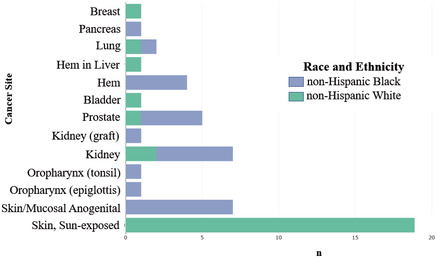

## 1 INTRODUCTION

Kidney transplantation is considered the most desirable treatment for patients with end‐stage renal disease (ESRD).^1^ Kidney transplantation significantly improves long‐term prognosis and quality of life in patients with ESRD compared to dialysis.^2^ However, kidney transplant recipients have a significantly increased risk of developing a malignancy and dying due to cancer compared to the general population.^3^, ^4^ The increased risk is attributed to the need for chronic immunosuppression that is required to preserve graft function.^5^, ^6^ Malignancies associated with oncogenic viral infections are especially high.^7^ The increased occurrence of cancer has been one of the main medical burdens in kidney transplant recipients.^8^ However, it is important that the providers should be aware of the risks associated with posttransplant malignancy in each patient to screen properly and provide medical management after a kidney transplantation.

Skin cancer, for example, is more common in lighter skin tone non‐Hispanic White (NHW) individuals compared to other races.^9^ However most data in US and European^10^ cancer registries is limited to melanoma types, making epidemiologic comparisons of risk in non‐melanoma skin cancer (NMSC) unavailable to guide prevention and screening efforts.^11^, ^12^

Different races and ethnicities from varying geographic regions have different cancer risks,^9^ and understanding the differences is critical to provide better posttransplant patient care. There are only a few studies focused on the differences among races and ethnicities by region in the United States.^4^, ^9^, ^13^, ^14^, ^15^ Herein, we retrospectively evaluated post‐kidney transplant malignancies in our transplant center in Eastern North Carolina with a focus on the differences of posttransplant skin cancer between NHW and non‐Hispanic Black (NHB) individuals.

## 2 MATERIALS AND METHODS

Consecutive adults, who received kidney transplantation at Vidant Medical Center/East Carolina University (ECU) in North Carolina between January 1, 2009, and December 31, 2015, were included in this retrospective study. We identified 538 adult kidney transplant recipients during these years. Any cancer diagnosis after transplantation surgery through February 18, 2020, was captured through detailed medical record review. We did not have adequate sample sizes to produce reliable estimates to investigate the intersections of all races and ethnicities. Therefore, only data from NHW and NHB were compared.

Of the 538 patients, 16 were excluded; of these, 11 were of Hispanic ethnicity and 5 were of Asian race. Of these individuals, only two, who were Hispanic developed a post‐kidney transplant cancer. An additional 81 patients were excluded because of a history of pretransplant malignancy. Two patients were excluded as clinical information was not available entirely. Thus, 439 recipients were included.

The study evaluated demography, time interval from transplant to the first cancer diagnosis, and histologic and immunohistochemical features. Race and ethnicity are based on the information reported in the electronic medical record (EMR).

The regarded cancer diagnoses were solely based on histopathological examination by an average of two pathologists, each in the Department of Pathology at ECU. If patients developed multiple cancers over time, only the first cancer diagnosis was included in the investigation.

Cancer‐containing tissues including skin specimens were formalin‐fixed, paraffin‐embedded, and sectioned 5 μm in thickness. Hematoxylin–eosin staining was performed in a standardized method. By immunohistochemistry, we examined rates of p16 positivity in the squamous cell carcinomas. P16 positivity is often considered a surrogate marker of integration of high‐risk human papillomavirus (HPV) DNA into the host genome.^16^ Positivity required intense, block‐like (diffuse) staining in the tumor cell nuclei. An automated immunohistochemistry system (Bond‐III; Leica Biosystems) was used for p16 detection, using the commercially available antibody p16 (6H12; Leica Biosystems).

Descriptive statistics, chi square tests and Fisher's Exact analyses were conducted in STATA StataCorp. 2021. *Stata Statistical Software: Release 17*: StataCorp LLC. This retrospective study was approved by ECU and Medical Center Institutional Review Board (IRB‐99‐0282) and authorization for waiver of consent was obtained.

## 3 RESULTS

### 3.1 Sample population characteristics

The baseline characteristics of 439 recipients are summarized in Table 1. Kidney transplant recipients were significantly more likely to be NHB, 312 (71.1%) versus 127 (28.9%) NHW; *p* < 0.001. At the time of transplant, no significant gender differences were seen, we had 239 males (54.4%) versus 200 females (45.6%), *p* = 0.17. The median age at the kidney transplant was 58.50 [51.50, 64.25] years of age with an age range of 17–82 years (Table 2).

**TABLE 1:** Baseline characteristics of the Kidney Transplant study population, January 1, 2009–February 18, 2020

Characteristic	Total	Cancer positive cases	Cancer negative cases	*p*‐value^a^
Study population				
*N*	439 (100%)	59 (13.4%)	380 (86.6%)	
Race				
White	127 (28.9%)	33 (55.9%)	94 (24.7%)	<0.001
Black	312 (71.1%)	26 (44.1%)	286 (75.3%)	
Sex				
Male	239 (54.4%)	37 (62.7%)	202 (53.2%)	0.17
Female	200 (45.6%)	22 (37.3%)	178 (46.8%)	
Age				
17–29	29 (6.6%)	3 (5.1%)	26 (6.8%)	0.025^b^
30–49	126 (28.7%)	8 (13.6%)	118 (31.1%)	
50–59	125 (28.5%)	21 (35.6%)	104 (27.4%)	
60–82	159 (36.2%)	27 (45.8%)	132 (34.7%)	

^a^ *p*‐Value estimated using chi‐square test/Fisher's exact test.

^b^ Fisher's exact test used because of small sample size in subgroups.

**TABLE 2:** Difference of sun‐exposed skin versus non‐sun‐exposed cancers between NH Whites and NH Blacks post‐kidney transplant recipients

Characteristic	NH Whites	NH Blacks	Total
Skin/mucosal cancer	25	7	32
Sex			
Male	15	2	17
Female	10	5	15
Age (median [IQR])	63.0 [54.0, 66.0]	40.0 [33.5, 52.0]	58.50 [51.50, 64.25]
Days to diagnosis (median [IQR])	1254.5 [729.0, 1899.5]	1747.00 [981.5, 2187.0]	1279.0 [837.5, 1973.5]
Cancer sites			
Sun‐exposed area	19	0	19
Non‐sun‐exposed anogenital area^a^	1	7	8
Unspecified skin site	5	0	5
Histologic diagnosis			
SCC (invasive)	14	3	17
SCC in situ	2	4	6
BCC	3	0	3
Unknown type	6	0	6

Abbreviations: BCC, basal cell carcinoma; IQR, interquartile range; NH, non‐Hispanic; SCC, squamous cell carcinoma.

^a^ Anogenital areas include vulvar, perianal, and anal.

### 3.2 New cancer cases

Of the overall sample (*N* = 439), 59 (13.4%) patients developed a post‐kidney transplant malignancy. Although our recipients were significantly more likely to be NHB, the overall number of new cancer cases in NHW was higher than that in NHB; 33 cancers among 127 NHW (55.9%) and 26 cancers among 312 NHB (44.1%). Of these, males were more affected, 37 (62.7%), than 22 (37.3%) females. The group aged 50 years and older had significantly more cancers than those under age 50; 46 (82.1%) versus 10 (17.9%), *p* = 0.003.

### 3.3 Cancer site and type

**FIGURE 1:**
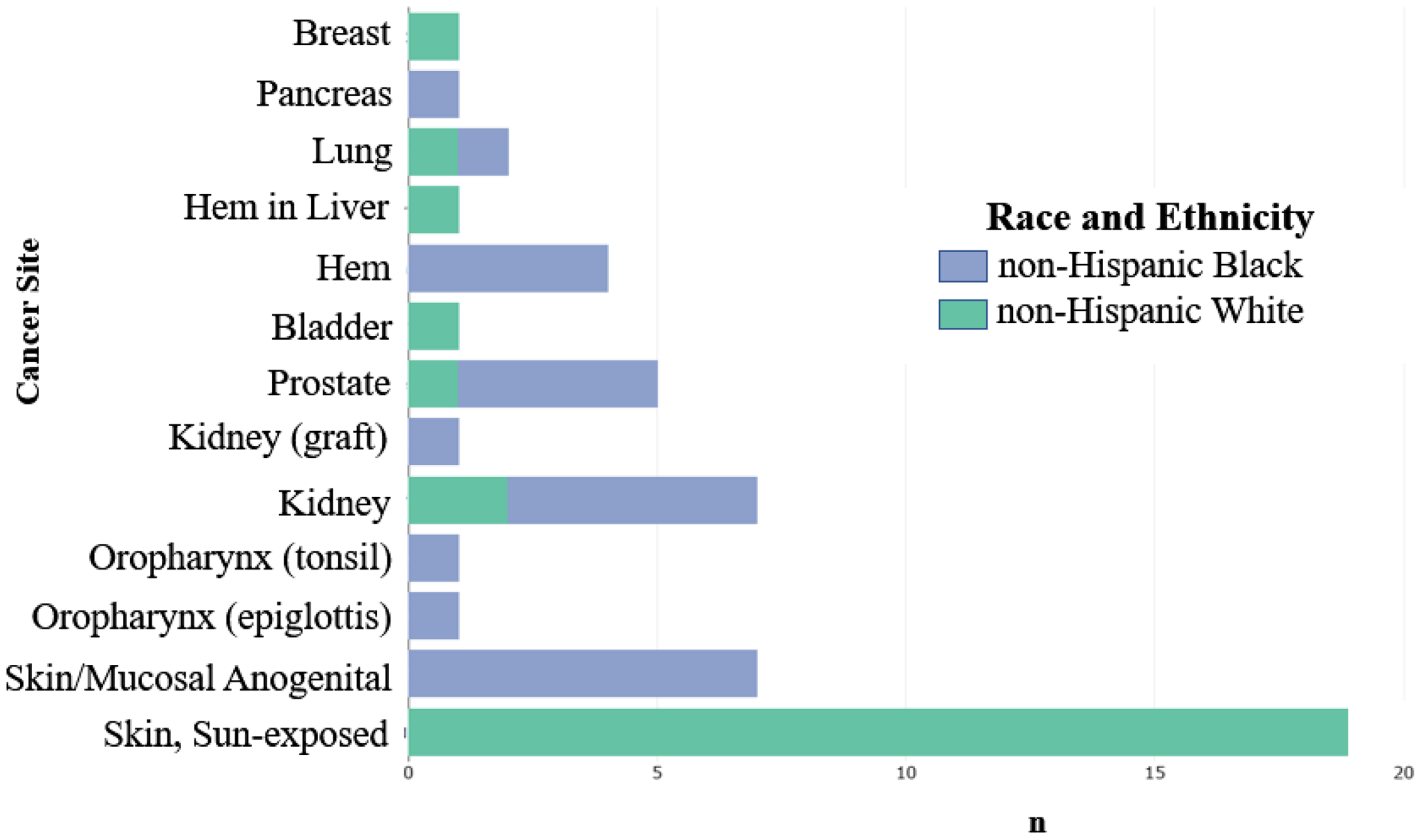
Summary of the posttransplant cancer sites in kidney transplant recipients by race and ethnicity.

**FIGURE 2:**
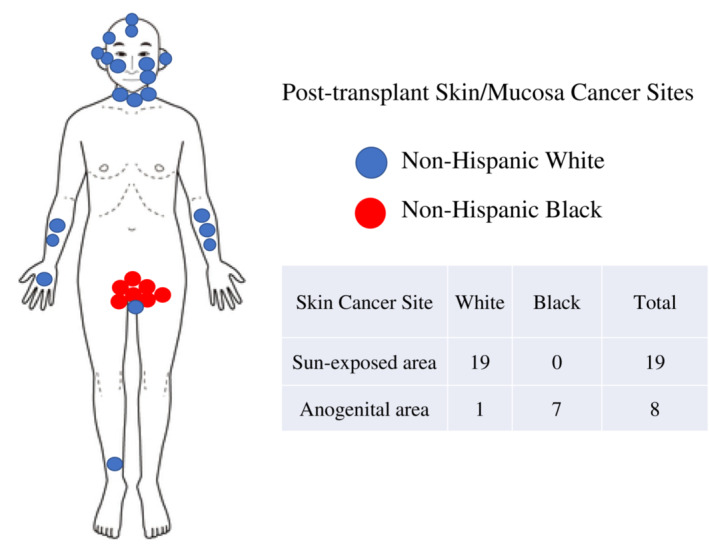
Differences in non‐sun‐exposed and sun‐exposed skin/mucosa cancer sites by race and ethnicity.

Figure 1 summarized cancer sites. Skin malignancies on sun‐exposed regions of the body were the most common followed by non‐sun‐exposed genital/mucosa skin and then kidney (Figure 2). The highest type prevalence was non‐melanoma squamous cell carcinoma on sun‐exposed areas in NHW. These were frequently associated with solar elastosis, histologic evidence of sun‐damaged skin. Genital cancers were located on the vulva, anal, or perianal regions. Each of these were squamous cell carcinomas with a high prevalence of p16 positivity, 83.3% (five out of six cases tested); p16 is a biomarker consistent with the presence of HPV infection. In sun‐exposed squamous cell carcinomas p16 positivity was 57.1% (four out of seven cases tested) (Data are not shown).

The most common histotype on skin/mucosa was squamous cell carcinoma (23 cases), followed by basal cell carcinoma (3 cases). Basal cell carcinoma was only seen on sun‐exposed skin in NHW. Malignant melanoma, Merkel cell carcinoma or Kaposi sarcoma were not observed in this study.

### 3.4 Skin/mucosa cancer racial and ethnic differences

We found striking racial and ethnic differences in sun‐exposed skin versus non‐sun‐exposed skin cancer (Figure 2). Among 25 skin cancers in NHW, the affected skin primary sites were available in 19 cases, and all except 1 developed on sun‐exposed areas such as head, cheek, ear, neck, forearm, and hand (Table 2). In contrast, seven cases of skin/mucosa cancer in NHB developed exclusively at genital areas such as perianal, anal, or vulvar regions. Skin cancers on sun‐exposed areas occurred more often in older individuals in NHW than those in genital skin/mucosa cancers in NHB; 63.0 years versus 40.0 years.

### 3.5 Mortality

Of the total transplant population (*N* = 439) in our study, 94 (21.4%) died between date of transplantation and February 18, 2020. (None of the deaths were attributed to COVID 19 on the death certificate.) Of note, one patient, a 57‐year‐old NHB female, developed and died from a previously non‐diagnosed, anal squamous cell carcinoma.

## 4 DISCUSSION

Our overall new cases in posttransplant cancers in adult kidney transplant recipients was significantly higher in NHW than that in NHB. Largely, this is attributed to new sun‐exposed skin cancers in NHW. In many of these transplant recipients, a clinically undetectable precancerous precursor lesion likely existed pretransplant. With the immunosuppression associated with the transplantation, the precursor lesions were able to blossom into clinically apparent frank malignancy. However, most cancer registries lack data on NMSC, and malignancies related to HPV infection, limiting surveillance and epidemiologic comparisons of cancer risks to guide posttransplant screening and management guidelines. In fact, with regard to mortality, one person died as a result of a previously undiagnosed squamous cell carcinoma of the anus. Our data suggest comprehensive posttransplant screening of the anogenital tract in recipients of all gender, race, and ethnicity.

Lighter skin tone individuals, which include NHW, have a significantly higher risk of NMSC and that risk elevates significantly in transplant recipients. One study that examined posttransplant recipients showed that NHW had a 22‐fold higher rate of skin cancer incidence compared to NHB.^9^ Another investigation found that the cancer incidence was comparable in NHW and NHB, although squamous cell carcinoma and basal cell carcinoma were more common in NHW, and Kaposi sarcoma was more common in NHB.^14^

We identified striking differences of skin/mucosa cancer sites between races. Skin cancers in NHW developed exclusively on sun‐exposed areas such as head, cheek, ear, neck, forearm, and hand; only one was detected on a genital area. On the other hand, NHB developed skin cancer exclusively in the anogenital area including vulva, anal or perianal regions. This finding is similar to one previous report of posttransplant genital skin cancers which occurred in an urban setting in the northeastern U.S.^17^ In contrast, our study population is from the rural southeastern US where lifetime sun exposure is more likely.

**FIGURE 3:**
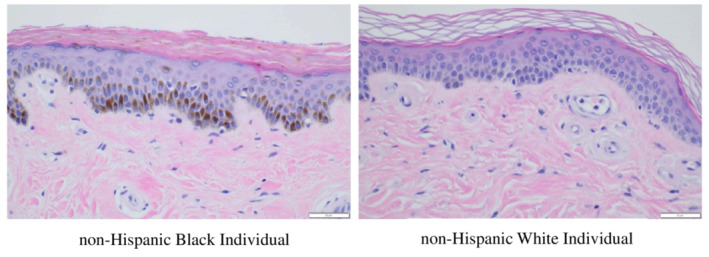
Normal histological differences in skin tone between lighter skinned non‐Hispanic White (NHW) and darker skin non‐Hispanic Black (NHB). These side‐by‐side histologic images of normal skin demonstrate that compared to NHWs, NHBs have markedly greater amounts of the solar‐protective protein melanin at the epidermal‐dermal junction (hematoxylin and eosin stains).

Sun exposure (ultraviolet rays) is an important risk factor for lighter‐skinned NHW as NHB are more protected from sun damage due to more deeply pigmented skin and higher melanosomes levels.^18^ In Figure 3, we show normal histological differences in skin tone between lighter skinned NHW and darker skin NHB. Although the published data is somewhat variable, it clearly appears that the density of melanocytes in the epidermis is not different among races. Rather, cytoplasmic melanosomes vary in their sizes, distribution, and arrangements.^18^ Further, the type of pigment proteins may differ in their quantity and protective properties.^19^

### 4.1 Genital infections—p16 and HPV

A well‐recognized fact is that immunological control of oncogenic viral infections is impaired, resulting in more common posttransplant cancers like Kaposi sarcoma and HPV‐related cancers. Squamous cell carcinoma arising in a genital area is frequently associated with preceding high‐risk HPV infection.^20^, ^21^, ^22^ In cervical and genital squamous cell carcinoma, p16 is proposed as a biomarker for transforming HPV infections.^23^ It is not clear why NHB in our study have specifically higher cancer cases in the genital area. One possible explanation is a greater proclivity in reactivation of existing high‐risk HPV infection by immunosuppression in NHB. This notion is supported by high prevalence of p16 positivity in genital cancer of NHBs in our study.

Although the social determinants of health are well‐known factors that increase cancer risk, some studies show that some differences might be related to infection of different HPV genotypes among races and ethnicities.^24^, ^25^, ^26^ Recent studies demonstrate significant racial differences between Black and White individuals in HPV genotype prevalence in uterine cervical dysplasia. Black individuals undergoing screening for cervical cancer were significantly less likely to be HPV16 positive and more likely to be positive for other high‐risk 12 HPV types compared to White individuals.^27^, ^28^, ^29^

In contrast to the direct relationship between HPV and p16 positivity in genital squamous cell carcinomas, the significance of p16 positivity in posttransplant squamous cell carcinoma on sun‐exposed skin should be interpreted cautiously. We found that p16 was positive in 57.1% of cases; however, this may not be related to infection by high‐risk HPV which is rare in cutaneous cancers in general. It is recognized that p16 positivity in some squamous cell carcinomas, that occur in the head and neck, esophagus, and lung, are unlikely due to high‐risk HPV infection.^30^ Although one publication reported that 46.2% of squamous cell cancer in immunosuppressed patients was positive for high‐risk HPV in association with diffuse p16 expression,^31^ the other showed that p16 expression in esophageal squamous cell carcinomas was associated with retinoblastoma (Rb1) protein loss but not HPV infection.^32^ Thus, it is controversial whether high‐risk HPV is involved in posttransplant squamous cell carcinoma in sun‐exposed skin. Further evaluation for the significance of p16 positivity is warranted.

Rather, posttransplant squamous cell carcinomas on the sun‐exposed skin is at least partially caused by a β‐HPV infection as in epidermodysplasia verruciformis type or cutaneous type (HPV 5 and 8) which are different from high‐risk HPV such as HPV16 and 18.^31^, ^32^, ^33^ Mechanistically, the E6 proteins of β‐HPV induces degradation of proapoptotic BAK and leads to inhibition of ultraviolet ray‐induced apoptosis.^34^ The more direct evidence that β‐HPV causes cutaneous squamous cell carcinoma was provided by a transgenic mice model; keratinocyte‐specific expression of HPV8 E6 protein was involved in squamous cell carcinoma in those transgenic mice.^35^

### 4.2 Microbiome

Epidemiologic evidence supports the association of HPV in the cervical vaginal microbiome with an increased risk of inflammation and HPV progression to cervical cancer.^36^, ^37^, ^38^ The composition of the vaginal microbiome may differ between White and Black individuals.^39^, ^40^ The effects of the skin microbiome on cancer progression, despite being the largest body organ, is less studied. The ecology of the skin surface is colonized by a diverse milieu of microorganisms that are highly variable.^39^ The host environment and topographical location where one lives and works influence the microbiota and, in turn, the cutaneous innate and adaptive immune responses.^40^, ^41^ Emerging evidence in the microbiome and the effect on the immune system creates a plausible association with an increase in cancer incidence posttransplant worthy of further study.

One limitation in this study is that racial and ethnic reporting is limited to that reported in the EMR, and we suggest it as a proxy for lighter and darker skin tones. We also recognize that race is a co‐created social construct, not a biological variable.^42^ However, biological differences secondary to lighter skin tone in NHW or the oncogenic type of HPV virus are suggested.^19^, ^26^, ^28^ Second, we lacked access to analyze sociocultural contributors.^43^ There are potentially six missing skin cancer reports due to outside community referrals, five missing cancer sites, and the cancer diagnosis dates were not included. Thus, this data was not incorporated into our medical record system. Despite that, we do not believe that aggressive types of skin cancer such as malignant melanoma or Merkel cell carcinoma were missed as these were likely to be referred to our facility to further treatments.

In conclusion, transplant medicine has been growing continuously and prevention of posttransplant malignancy with comprehensive skin inspection, including anogenital areas, is crucial. Skin cancer was the most common malignancy, and squamous cell carcinoma is the most common histologic type. In addition, 95% of known skin cancer sites in NHW developed on sun‐exposed areas; in contrast, 100% developed on non‐sun‐exposed, anogenital areas in NHB individuals. Different oncogenic mechanisms involving ultraviolet rays, possible different HPV type infections, the skin microbiome, potential genetic or biological differences among races, and the types of immunosuppression used all may play a role. This study indicates that screening with attention to risk stratification for kidney transplant recipients based on cancer site, cancer type, and potentially race and ethnicity may aid in the early detection of malignancies.

## AUTHOR CONTRIBUTIONS

**Kotaro Takeda:** Conceptualization (equal); data curation (equal); formal analysis (equal); investigation (equal); methodology (equal); writing – original draft (lead); writing – review and editing (lead). **Carolann Risley:** Formal analysis (equal); funding acquisition (equal); investigation (equal); methodology (equal); writing – original draft (lead); writing – review and editing (lead). **Aisha Kousar:** Data curation (equal); investigation (equal). **Kimberly P. Briley:** Data curation (equal). **Karyn Prenshaw:** Investigation (equal). **Rajesh Talluri:** Formal analysis (equal); writing – review and editing (supporting). **Kim R. Geisinger:** Conceptualization (equal); investigation (equal); methodology (equal); supervision (equal); validation (equal); writing – original draft (lead); writing – review and editing (equal). **Lorita M. Rebellato:** Conceptualization (equal); data curation (equal); investigation (equal); project administration (equal); supervision (equal); writing – original draft (equal); writing – review and editing (equal).

## FUNDING INFORMATION

This work was supported by the Mississippi Center for Clinical and Translational Research Center – National Institute of General Medical Sciences of the National Institutes of Health under Award Number 1U54GM115428.

## CONFLICT OF INTEREST

The authors do not have any conflict of interests to disclose.

## DISCLAIMER

The views expressed in this manuscript are those of the authors and do not reflect the official policy of the Department of Defense or the US government.

## ETHICAL APPROVAL STATEMENT

The use of patient data was approved by the East Carolina University Brody School of Medicine Institutional Review Board for human studies and authorization for waiver of consent was obtained. All clinical and research activities are consistent with the Principles of the Declaration of Istanbul.

## Data Availability

Data that support the findings of this study are available from the corresponding author upon request.
